# The sentinels of coronary artery disease: heterogeneous monocytes

**DOI:** 10.3389/fimmu.2025.1428978

**Published:** 2025-02-26

**Authors:** Yanyu Chen, Daya Luo, Renzhuo Gao, Jinjing Wu, Xingpeng Qiu, Yang Zou, Yingchao Jian, Shuhua Zhang

**Affiliations:** ^1^ Jiangxi Cardiovascular Research Institute, Jiangxi Provincial People’s Hospital, The First Affiliated Hospital of Nanchang Medical College, Nanchang, China; ^2^ Tongji Hospital, Tongji Medical College, Huazhong University of Science and Technology, Wuhan, China; ^3^ School of Basic Medical Sciences, Jiangxi Medical College, Nanchang University, Nanchang, China; ^4^ Queen Mary College, School of Medicine, Nanchang University, Nanchang, China; ^5^ School of Basic Medical Sciences, Nanchang University, Nanchang, China; ^6^ Department of Radiology, Jiangxi Provincial People’s Hospital, The First Affiliated Hospital of Nanchang Medical College, Nanchang, China

**Keywords:** monocyte heterogeneity, atherosclerosis, inflammation, coronary artery disease, flow cytometry

## Abstract

Monocytes are heterogeneous immune cells that play a crucial role in the inflammatory response during atherosclerosis, influencing the progression and outcome of the disease. In the pathogenesis of atherosclerotic diseases, such as coronary artery disease (CAD), monocytes not only serve as the initial sensors of endogenous and exogenous pathogenic factors, but also function as intermediators that bridge the circulatory system and localized lesions. In the bloodstream, heterogeneous monocytes, acting as sentinels, are rapidly recruited to atherosclerotic lesions, where they exhibit a heightened capacity to respond to various pathological stimuli upon detecting signals from damaged vascular endothelial cells. Clinical studies have demonstrated that the heterogeneity of monocytes in CAD patients presents both diversity and complexity, varying across different disease subtypes and pathological stages. This review explores the heterogeneity of monocytes in CAD, focusing on alterations in monocyte subset numbers, proportions, and the expression of functional receptors, as well as their correlations with clinical features. Additionally, we propose strategies to enhance the clinical utility value of monocyte heterogeneity and outline future research directions in the field of CAD. With the widespread application of high-parameter flow cytometry and single-cell sequencing technologies, it is anticipated that a comprehensive understanding of monocyte heterogeneity in CAD will be achieved, enabling the identification of disease-specific monocyte subtypes. This could offer new opportunities for improving the diagnosis and treatment of CAD.

## 1 Introduction

Coronary artery disease (CAD) is a type of heart condition characterized by narrowing or blockage of the coronary arteries due to atherosclerosis, which subsequently leads to myocardial ischemia, hypoxia, or even necrosis (1–3).Although people have a better understanding of the pathogenesis of atherosclerosis, CAD remains the primary cause of mortality worldwide (4) and is affecting an increasing number of younger individuals compared to previous periods (5). The severity of CAD is closely related to the pathological progression of atherosclerosis. To grasp the whole picture of the disease and explore feasible new treatment strategies, the pathological mechanism of atherosclerosis has been extensively explored. At present, the “inflammatory response theory” proposed by Professor Ross (6) is generally accepted, which holds that atherosclerosis is a chronic inflammatory disease, and that a variety of immune cells, including monocytes/macrophages, lymphocytes, Neutrophils and Dendritic cells play import roles in the process of atherosclerosis. This chronic inflammation plays an important role in the whole process of atherosclerosis. In this process, the recruitment and activation of monocytes represent the initial event of atherosclerosis (7), which involves the initiation of inflammation, proceeds through the beginning and end of the inflammatory response, and plays a crucial role in regulating the progression and outcome of the disease.

Monocytes originate from bone marrow myeloid haematopoietic progenitor cells, mature in circulating blood, are recruited by local tissue inflammatory response signals, and participate in the inflammatory response (8, 9). Monocytes consist of three distinctly heterogeneous subpopulations, and the transition among them represents a gradual maturation process. Early in the 1990s, human monocytes were divided into classical monocytes (CD14^+^CD16^-^) and nonclassical monocytes (CD14^+^CD16^+^) according to the expression levels of the surface molecules CD14 and CD16. CD14, a coreceptor for Lipopolysaccharide, is a surface antigen that is preferentially expressed on monocytes/macrophages. It cooperates with other proteins to mediate the innate immune response to bacterial lipopolysaccharide, and to viruses. CD16 (also known as FcγRIII) is a receptor for the Fc portion of immunoglobulin G, and it is involved in the removal of antigen-antibody complexes from the circulation, as well as other responses (10). In the following two decades, many studies reported that CD14^+^CD16^+^ monocytes can be subdivided into intermediate monocytes (CD14^++^CD16^+^) and nonclassical monocytes (CD14^+^CD16^++^). In 2010, the Nomenclature Committee of the International Union of Immunologic Societies “officially” recognized and named 3 monocyte subsets: classical (CD14^++^CD16^−^), intermediate (CD14^++^CD16^+^), and nonclassical (CD14^+^CD16^++^) (11). In 2016, the European Society of Cardiology (ESC) Working Groups updated the nomenclature of 2010, suggesting that the numerical labels “Mon1” represents classical monocytes, “Mon2” represents intermediate monocytes, and “Mon3” represents nonclassical monocytes (12). Here, we use numerical labels to represent the 3 common subgroups of monocytes.

**Figure 1:**
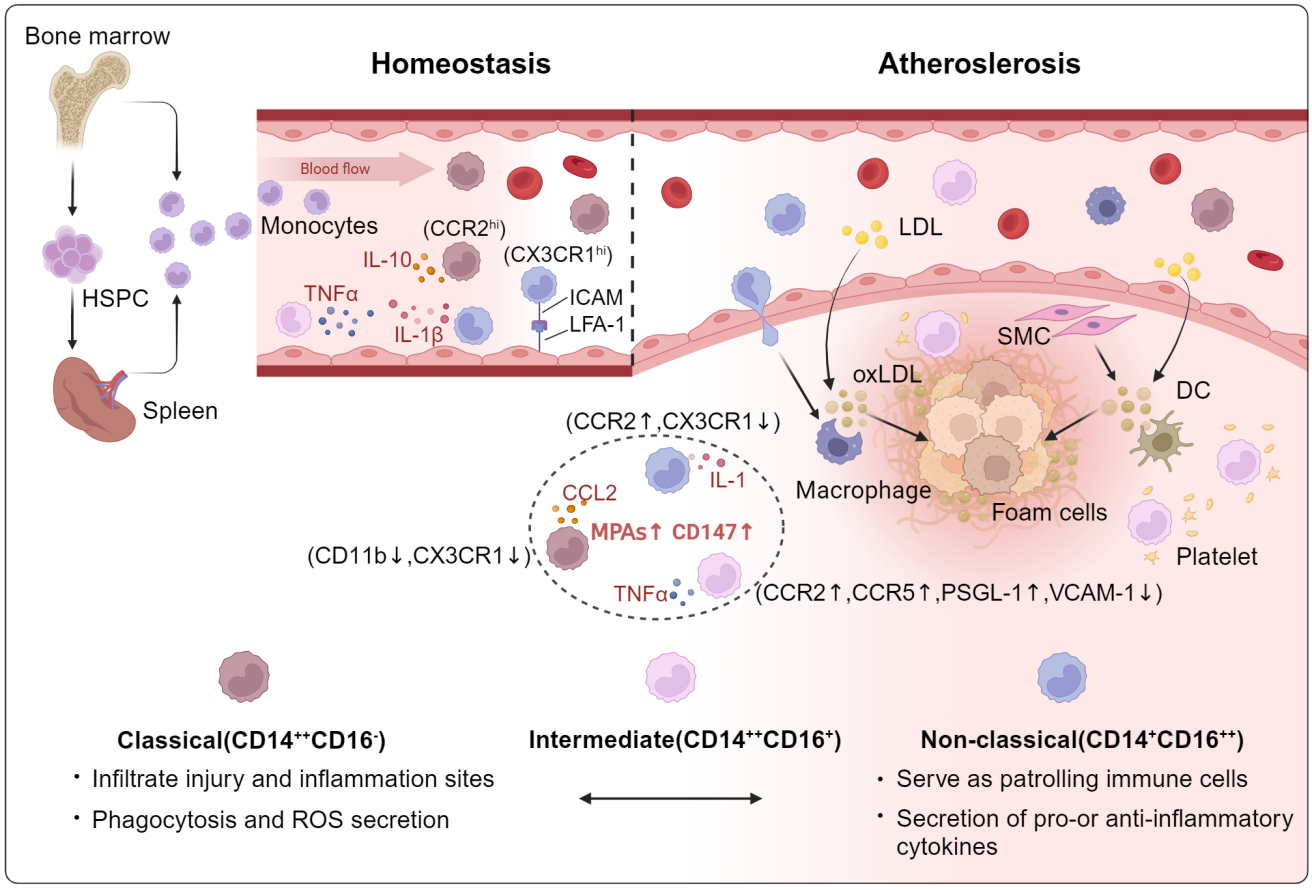
The role of circulating monocyte heterogeneity in Atherosclerosis. Mon1 is the great subtype of circulating monocytes, its main function is to respond to bacterial stimulation of phagocytosis and production of ROS. Mon1 cells were demonstrated to differentiate into Mon2, which in turn differentiated into Mon3 monocytes. In contrast to Mon1, Mon3 cells produce anti-inflammatory cytokines and proinflammatory cytokines, such as IL-10 and IFN-α, in response to bacterial and viral responses, respectively. Mon3 patrol the vascular system in a steady state to show the “patrol” function, which is helpful to maintain vascular integrity. Mon2 cells exhibit both Mon1 and Mon3 molecular phenotypes, and their functional characteristics often overlap. HSPC, hematopoietic stem progenitor cell; ROS, reactive oxygen species; LDL, low-density lipoprotein; SMC, smooth muscle cell; DC, dendritic cells.

The 3 subgroups of monocytes have significant functional heterogeneity, and the transition among them is a gradual maturation process. Mon1 is the main subtype of circulating monocytes. They are highly active in phagocytosis and the production of reactive oxygen species (ROS). In response to bacterial signals, Mon1 cells secrete inflammatory cytokines such as IL-6, IL-8, and IL-1β (13). Moreover, Mon1 monocytes were found to differentiate into Mon2 monocytes, which in turn differentiated into Mon3 monocytes (14). In contrast to Mon1, Mon3 cells produce anti-inflammatory cytokines and proinflammatory cytokines, such as IL-10 and IFN-α, in response to bacterial and viral responses, respectively (15). Mon3 cells patrol the vascular system in a steady state to show “patrol” function, which helps to maintain vascular integrity. Mon2 cells exhibit both Mon1 and Mon3 molecular phenotypes, and their functional characteristics often overlap. Mon2 can be quickly recruited to the lesion site to participate in the inflammatory response (16), which seems to have the strongest correlation with adverse cardiovascular events in CAD patients (**Figure 1**). Although the immunological functions of these 3 monocyte subgroups have been well studied, their specific roles in the development and progression of atherosclerosis remain to be further explored.

This article summarizes the clinical research status of monocyte heterogeneity in CAD by identifying correlations between peripheral blood monocyte heterogeneity and clinical characterization, imaging indices, and other hematological indices of CAD patients to investigate the potential role of monocytes in different types and stages of CAD and to establish a rational prospect for the future research direction of monocyte heterogeneity.

## 2 Stable coronary artery disease and monocyte heterogeneity

Stable Coronary Artery Disease (SCAD) is characterized by the progressive development of atherosclerosis in the coronary arteries, leading to vessel narrowing and insufficient blood supply to the myocardium. Compared to acute coronary syndrome (ACS), the clinical presentation of SCAD exhibits greater stability without acute exacerbation (17). Stable angina, a common manifestation of CAD, is precipitated by atherosclerotic plaques comprising a lipid-rich core encased by a fibrous cap., These plaques accumulate on the arterial walls over time, resulting in gradual luminal narrowing. This process diminishes blood flow to the myocardium, particularly during periods of increased demand, such as physical exertion or stress. The plaques instable angina typically exhibit greater stability, with a thicker fibrous cap that confers protection against rupture (6). However, factors such as cap thinning or increased inflammation may still contribute to plaque vulnerability, underscoring the significance of immune cells in CAD pathophysiology. The formation of atherosclerotic plaques involves lipid deposition and inflammatory responses, with monocytes playing a pivotal role in plaque progression. Monocytes infiltrate plaques, subsequently differentiating into macrophages that contribute to plaque instability (18).

Compared with those in healthy controls, the number and proportion of monocyte subsets in patients with CAD significantly differ. A study by Sturhan et al. (19) reported that the proportion of Mon1 cells to total monocytes in patients with SCAD was lower than that in healthy controls, whereas the proportions of Mon2 and Mon3 monocytes were greater. Czepluch et al. (20) demonstrated that the percentage of Mon1 monocytes among the total monocytes of patients with SCAD was elevated, whereas Mon3 was reduced compared with that in the healthy group. The results of these 2 studies are not consistent, which may be related to the small sample size and the presence of confounding factors among the subjects. However, these preliminary findings suggest that the number and proportion of monocyte subsets indeed change in the early stage of CAD. However, the functions of monocyte subsets are unclear.

Studies on functional changes in monocytes have focused mainly on the expression of functional receptors on the surfaces of monocytes and their interactions with other cells or cytokines. Monocyte platelet aggregate (MPA) has been shown to be a sensitive early indicator of platelet activation *in vivo* and may directly regulate vascular inflammation, atherosclerosis progression, and thrombosis formation (21). Czepluch et al. (20) found that the levels of MPA in 3 monocyte subsets in patients with SCAD were greater than those in healthy controls, whereas the expression rate of the platelet chemokine receptor CCR5 decreased. Furthermore, Mon2 is the majority subset of CCR5 expression in both the healthy control group and the CAD group. Toll-like receptor 4 (TLR4) is a receptor for exogenous lipopolysaccharide (LPS) and endogenous heat shock proteins, which play important roles in innate immunity and the inflammatory response. TLR4 has been shown to be involved in monocyte activation in patients with accelerated atherosclerosis (22). Mon2 is the subgroup with the highest TLR4^+^ expression in the circulating monocytes of patients with CAD. The proportion of TLR4^+^ Mon2 cells in patients with unstable plaques was greater than that in patients with stable plaques and was positively correlated with the content of matrix metalloproteinase 9 (MMP-9) in the blood, as well as plaque vulnerability (23). Other monocyte activation markers, such as CD11c, have also been confirmed to be selectively and highly expressed on Mon3 in patients with CAD, and the expression level is related to endothelial dysfunction (24). Studying the correlation between monocyte heterogeneity and clinical phenotype is of great significance and will further expand the clinical value of monocyte heterogeneity.

Monocytes are known to be active throughout the formation of atherosclerotic plaques, and the specific stages and characteristics of plaques need to be detected by imaging equipment. Manabu Kashiwagi et al. (25) estimated plaque vulnerability in patients with stable angina pectoris (SAP) via 64-slice multidetector computed tomography (MDCT) and evaluated its relationship with peripheral blood heterogeneity. Monocytes were divided into CD14^+^CD16^-^ and CD14^+^CD16^+^ groups, and the results revealed that the increase in the proportion of CD14^+^CD16^+^ monocytes was positively correlated with plaque vulnerability. Another study (26) confirmed that an increase in the proportion of CD14^+^CD16^+^ cells was positively associated with the development of future adverse cardiovascular events. The study by Ozaki et al. (27) reached a similar conclusion; according to the results of coronary angiography, patients were divided into 3 groups: the no coronary artery disease group, single-vessel disease group (SVD), and multivessel disease group (MVD). The counts and proportions of CD14+CD16+ monocytes in patients with MVD were greater than those in patients with SVD or without CAD and were positively correlated with the Gensini score indicating the severity of coronary artery disease. These studies have consistently shown that the proportion and number of CD14+CD16+ monocytes in the peripheral blood of SAP patients may be used to evaluate the risk of future cardiovascular events. However, they have the common limitation that CD14+CD16+ monocytes contain both mon2 and mon3 cells as previously defined. The results may be more accurate if monocyte subpopulations are distinguished into three subsets as recommended by the ESC.

## 3 Acute coronary syndrome and monocyte heterogeneity

ACS is triggered by the rupture or erosion of atherosclerotic plaques, leading to thrombosis that partially or completely occludes the coronary arteries, resulting in myocardial ischemia and damage. ACS includes ST-segment elevation myocardial infarction (STEMI), non-ST-segment elevation myocardial infarction (NSTEMI), and unstable angina pectoris (UAP) (28). Plaque rupture exposes thrombogenic material to the bloodstream, initiating thrombus formation and partial or complete coronary occlusion, which subsequently leads to ischemia. Ischemic myocardium releases damage-associated molecular patterns (DAMPs), activating the immune system and exacerbating inflammation (29). Although reperfusion therapy restores blood flow, it may induce reperfusion injury, characterized by oxidative stress, calcium overload, and inflammatory activation, which further exacerbates myocardial damage (30).

The relationship between monocyte heterogeneity and ACS has attracted considerable attention. P-selectin glycoprotein ligand-1 (PSGL-1) participates in monocyte activation in patients with thrombosis (31). In patients with ACS, the expression of PSGL-1 in Mon2 cells was greater than that in Mon1 or Mon3 cells (32). Shantsila et al. (33) reported that the proportion of Mon2 was independently associated with the activity of plasminogen activator inhibitor-1 (PAI-1) in patients with ACS. We reviewed clinical studies related to monocyte heterogeneity in this field according to the classification of ACS. Given that most of the literature on acute myocardial infarction (AMI) does not distinguish between STEMI and NSTEMI, AMI-related studies are described first.

### 3.1 Acute myocardial infarction

AMI occurs often accompanied by disturbances in immune homeostasis, including significant changes in the phenotype and function of various immune cells such as monocytes, neutrophils, T cells, and dendritic cells (34). Compared with SCAD, the monocyte heterogeneity in AMI is more complex, and the number and proportion of monocyte subsets are altered. In the study by Ozaki Y et al. (22), the proportion of CD14+CD16+monocytes was found to be lower in AMI patients than those in the SAP or UAP group. Additionally, the ratio of Mon1 to total monocytes was lower in AMI patients than that in healthy controls, whereas the ratios Mon2 and Mon3 were greater (19). Another study (35) revealed that AMI could induce a transient increase in the absolute number of Mon1 monocytes and a continuous increase in Mon2 and Mon3 monocytes following ischemic injury. These findings demonstrated that during myocardial ischemia, there is a substantial increase in circulating monocytes to be recruited to the lesion site when the organism is under acute stress, and that Mon1, as the largest subpopulation, exhibited the most significant transient changes in numbers. Subsequently, to adapt to the pathological environment, the majority of Mon1 differentiated into Mon2 and Mon3, which balance the inflammatory response and facilitate vascular repair, resulting in an increase in Mon2 and Mon3.

The heterogeneity of monocytes in patients with AMI is also reflected in changes of monocyte functions, such as phagocytosis, migration, secretion, and adhesion and the expression of receptors that reflect their activity. Compared with those in healthy volunteers, the phagocytosis and migration activities of monocytes in patients were enhanced 3 days after AMI (35). Extracellular matrix metalloproteinase inducer (EMMPRIN, CD147) is considered the main regulator of monocyte MMP-9 in patients with AMI. Its expression on the surfaces of the 3 monocyte subtypes is increased compared with that in patients with SCAD (19). Similar changes can be observed in the expression of TLR4 (22). CD11c/CD18 is a functional receptor expressed on the inflammatory subset of monocytes, and is upregulated in apolipoprotein E-deficient mice with dyslipidaemia induced by a high-fat diet. Foster et al. (36) found that the adhesion efficiency of Mon2 in patients with AMI was sevenfold greater than that of other subsets and double that in healthy subjects, which was related to the increased expression of CD11c/CD18 on Mon2. These findings suggest that the surface receptor molecules that affect the adhesion of monocytes to endothelial cells warrant greater attention in the acute stage of the disease.

In clinical research on AMI, monocyte heterogeneity is often combined with other clinical indicators, which has important guiding value for the diagnosis, treatment, and evaluation of the disease. Ikejima et al. (37) selected 29 patients with primary AMI who were successfully treated with primary percutaneous coronary intervention (PCI) to analyze the relationship between changes in monocyte subsets and the coronary flow velocity reserve (CFVR). ΔCFVR is defined as the difference between the CFVR values on Day 4 and Day 7, and is used to reflect the degree of myocardial salvage. The results revealed a significant negative correlation between Mon1 count and ΔCFVR in patients with AMI, and a similar result was observed in the study of Ikuko Teraguchi et al. (38), suggesting that the effects of physical factors such as haemodynamics, including fluid shear stress, on monocyte heterogeneity warrant attention. Another study by Teraguchi et al. (39) divided AMI patients into a plaque rupture group and an unruptured group by optical coherence tomography (OCT). The number of Mon2 cells in patients with plaque rupture was significantly greater than that in patients without plaque rupture and was positively correlated with the mean amplitude of glycaemic excursion (MAGE). which was measured by a continuous glucose monitoring system 7 days after the onset of AMI. MAGE is an indicator of glycemic variability and primarily used to assess the stability of glycemic in patients with diabetes. This study showed for the first time that MAGE was significantly associated with plaque rupture in patients with primary AMI and suggested that dynamic glucose fluctuations may be associated with plaque rupture, potentially through the preferential increase in Mon2 monocyte levels. The above clinical studies have shown that the inflammatory activity of monocytes is activated, however, the heterogeneity of monocytes is diverse in patients with AMI, which may be related to the lack of a clear AMI classification in the patients.

### 3.2 Non-ST-elevation myocardial infarction

AMI is traditionally divided into 2 types on the base of the manifestations observed on an electrocardiogram: STEMI and NSTEMI. Although STEMI and NSTEMI have similar risk factors and aetiologies, they differ in pathophysiology, epidemiology, and severity (40). In most situations, AMI is caused by the rupture of atherosclerotic plaques or erosion of the coronary artery endothelium, which is triggered by inflammation followed by thrombosis. STEMI occurs when a thrombus completely blocks the coronary artery, whereas NSTEMI occurs when a thrombus does not completely block the coronary artery (41). The varying degrees of occlusion may lead to different vascular microenvironment, which in turn affects monocyte heterogeneity. Therefore, we reviewed the heterogeneity of monocyte in the STEMI and NSTEMI patients separately.

Although the absolute number of all monocyte subsets in NSTEMI patients, similar to that in STEMI patients, is greater than that in control patients (42, 43), the heterogeneity of monocyte in NSTEMI patients cannot be overlooked. The recruitment of monocytes into the vicinity of inflammatory artery tissue is important for aggravating the pathologic progression of atherosclerosis. During this process, adhesion factors, chemokines, and integrins secreted by monocytes play important roles. For example, CD11b and CD11c promote monocyte adhesion to endothelial cells (44); the chemokine receptor CX3CR1 plays a key role in monocyte migration (45); the human leukocyte antigen DR (HLADR) affects the proinflammatory or antigen presentation ability of monocytes (46), and their expression patterns on circulating monocytes in patients with NSTEMI all reflect the characteristics of the disease. Hernandez et al. (47) reported that the expression of CD11c and CX3CR1 in all monocytes in the NSTEMI group was greater than that in the healthy control group, and Mon2 and Mon3 exhibited substantial increases in CD11c expression of more than twice that in the healthy group. The expression of VLA-4 was greater than that in the CAD and healthy groups only in Mon2. These results demonstrate the activation of the inflammatory chemotaxis potential of monocytes in patients with NSTEMI. Math P. G. Leers et al. (43) showed that the expression of CD11b in the peripheral blood monocytes of patients with NSTEMI was increased, whereas the expression levels of HLA-DR in Mon2 and Mon3 and CX3CR1 in Mon1 and Mon2 were decreased compared with those in non-ACS patients. The expression of TLR4 in monocytes has also been discussed in NSTEMI. Tapp et al. (48) recruited 50 patients with STEMI, 48 patients with NSTEMI, and 40 CAD patients to monitor changes in monocyte heterogeneity on Days 1, 3, 7, and 30 after myocardial infarction. Three monocyte subsets were defined as CD14^++^CD16^-^CCR2^+^, CD14^++^CD16^+^CCR2^+^, and CD14^+^CD16^++^CCR2^-^, and the expression of TLR4 in monocytes was measured. The results revealed a significant increase in the number of TLR4^+^CD14^++^CD16^+^CCR2^+^ monocytes in NSTEMI patients compared with CAD patients, and the number of TLR4^+^CD14^++^CD16^+^CCR2^+^ was positively correlated with the plasma interleukin-6 (IL-6) level. The authors specifically noted that the increase in TLR4^+^CD14^++^CD16^+^CCR2^+^ subset was attributed to an increased monocyte subset count rather than TLR4 expression in monocytes. In general, the difference in the expression of these functional receptors reflects the proinflammatory state of monocytes in NSTEMI. Therefore, the use of functional receptors as target molecules to reduce the migration of proinflammatory monocytes to myocardial tissue may be a new therapeutic strategy.

In addition, the number of monocyte subsets in NSTEMI patients is related to clinical indicators. Arslan et al. (42) reported that the peak value of Mon1 was directly proportional to the peak levels of CK and CK-MB in patients with NSTEMI on the 7th day after onset. The peak value of Mon2 was proportional to the Gensini score. Therefore, the heterogeneity of Mon1 and Mon2 cells among circulating monocytes may be more significantly associated with NSTEMI patients than with healthy controls and other types of CAD patients.

### 3.3 ST-elevation myocardial infarction

The difference in monocyte heterogeneity between STEMI and NSTEMI patients was significant. Compared with the NSTEMI or SAP group, patients with STEMI had higher peak levels of Mon1 and Mon2 within 7 days after onset but reached the peak later (42). Similarly, Shantsila et al. (49) also reported that the absolute numbers of Mon1 and Mon2 cells in STEMI patients were greater than those in healthy controls and SCAD patients. Moreover, compared with that in ACS-negative patients, the absolute number of monocytes clearly and significantly increased in patients with STEMI, and this increase was observed in all subtypes of monocytes (43). In addition, circulating monocyte heterogeneity has been demonstrated to dynamically change during the onset of STEMI. Bosch et al. (50) reported that the number of Mon1 and Mon2 of STEMI patients who experienced initial onset and completed PCI within 12 hours decreased continuously for 7 days, whereas Mon3 cells showed the opposite trend. The results of this study differ from those of other researchers, possibly because of the small sample size and the absence of a control group. Recently, Mohamed et al. (51) recruited 100 STEMI patients who underwent primary PCI and analyzed dynamic changes in circulating monocyte heterogeneity within 24 hours after presentation. The results verified that an increased number of circulating Mon3 cells and their subsets expressing CCR2, CD42, and CD11b could be important predictors of clinical events during long-term follow up in STEMI patients. Although the results of these studies are not completely consistent and may be related to sampling at different time points from the initial onset of disease, they all suggest that monocyte heterogeneity may serve as a significant marker to assess STEMI progression.

Distinct immunophenotypic patterns of monocyte heterogeneity are observed in patients with STEMI versus those with NSTEMI. Compared with that in the ACS-negative group, the expression of CD11b decreased in all monocytes, especially in Mon1 monocytes, and the expression of CX3CR1 was reduced in all monocytes. Notably, the level of CX3CR1 decreased in Mon3, which was present in only the STEMI group (43). Eduard et al. (52) divided monocytes into KDR^+^ angiogenesis and CXCR4^+^ vascular repair potential according to the expression of functional receptors. Interestingly, the numbers of both CXCR4^+^ and KDR^+^ monocytes increased after myocardial infarction, and their correlation with CD14^++^CD16^+^CCR2^+^ monocytes was more significant. These results suggest that CD14++CD16+CCR2+ cells play the most prominent role in myocardial infarction repair. Other functional receptors on the surfaces of monocytes, such as intercellular adhesion molecule-1 receptor (ICAM-1r), vascular cell adhesion molecule-1 receptor (VCAM-1r), and interleukin-6 receptor (IL-6r), play important roles in regulating monocyte inflammatory activity and migration to damaged tissue, and they have also been confirmed to be altered in patients with STEMI. The expression of VCAM-1r in 3 subsets of STEMI patients decreased at admission, and the expression of VCAM-1r on Mon1 was positively correlated with the plasma IL-1β level. The level of IL-6r was significantly lower in Mon2 of patients with STEMI than that of SCAD patients and gradually increased on Mon1 and Mon2 cells, with no change in ICAM-1r during follow-up (53). The decreased expression of IL-6r and VCAM-1r in circulating monocytes involved in STEMI inflammation may represent a regulatory feedback mechanism aimed at rebalancing the typical inflammation caused by the selective homing of monocytes with high expression of receptors after AMI.

Monocyte heterogeneity is closely related to the prognosis of patients with STEMI. Dectin-1, a pattern recognition receptor, is expressed on activated myeloid cells and is essential for the regulation of immune homeostasis. Fan et al. (54) found that Dectin-1 expression in Mon1 and Mon2 in STEMI patients was significantly greater than that in the control group and was positively correlated with the severity of cardiac insufficiency. The data of 71 STEMI patients who underwent bare-metal stent (BMS) implantation for a 9-month follow-up revealed that the number of CD14^+^CD16^+^CX3CR1^+^ subsets in patients with restenosis was significantly greater than that in patients without restenosis. The number of CD14^+^CD16^+^CX3CR1^+^ cells seems to be able to predict the prognosis of patients after treatment, which provides a new direction for assessing the risk of late instent restenosis (55). A few years later, the role of CD14^++^CD16^+^CCR2^+^ monocytes was confirmed in another study. Their absolute number is an independent predictor of major adverse cardiovascular events (MACEs) in patients with STEMI (56). Tsujioka et al. (57) evaluated the correlation between monocyte heterogeneity and microvascular occlusion (MVO) within 8 days after stent implantation in patients with primary STEMI and reported that the peak level of CD14^+^CD16^-^ monocytes in patients with MVO was significantly greater than that in patients without MVO. In summary, circulating monocyte heterogeneity could be used to evaluate the prognosis and risk of adverse cardiovascular events associated with STEMI.

### 3.4 Unstable angina pectoris

UAP is a clinical condition falling within the ACS continuum and is defined as myocardial ischaemia at rest or on minimal exertion in the absence of acute cardiomyocyte necrosis. The development of high-sensitivity troponin assays has increased the detection rate of myocardial necrosis, increasing the frequency of NSTEMI diagnosis at the expense of UAP (58). In contrast to stable angina, atherosclerotic plaques in patients with unstable angina exhibit a thinner fibrous cap, a larger lipid core, and more infiltration of inflammatory cells. The combined effects of these three factors increase the instability of the plaque (6). In fact, patients with UAP tend to be younger than those with NSTEMI but have a higher prevalence of most cardiovascular risk factors and more advanced coronary artery disease. These patients have a nonnegligible cardiovascular risk (59). These clinical research results have shown that patients with UAP also have a unique heterogeneous monocyte expression pattern.

With respect to the proportion and number of monocyte subsets, patients with UAP had significantly greater proportions and counts of Mon2 than that did the healthy control group, similar to patients with STEMI (60). The absolute number of Mon1 cells in the UAP group was lower than that in the NSTEMI group (43). Compared with the AMI group, the UAP group presented a clear and significant increase in the percentage of CD14^+^CD16^+^ monocytes (22). Conversely, Mon2 and Mon3 cells were more abundant in patients with UAP than those in patients with SCAD (61). In general, circulating Mon2 cells are more active in patients with UAP. Compared with STEMI patients, patients with UAP had significantly greater expression of CD11b on Mon1, whereas the expression of CD11b on Mon2 and Mon3 cells was lower than in the NSTEMI group (43). Ozaki Y et al. (32) investigated the relationship between the expression of PSGL-1 on monocyte subsets and thrombus formation via frequency domain optical coherence tomography (FD-OCT) in patients with ACS. They enrolled 100 individuals, including subjects with AMI, UAP, and SAP and control subjects. Circulating monocytes were divided into 3 groups: Mon1, Mon2, and Mon3. The results revealed that Mon2 expressed PSGL-1 more frequently in the UAP group than in the control group but more infrequently than in the AMI group, with no significant difference between the UAP group and the SAP group. Kashiwagi M et al. (22) by used flow cytometry to measure the expression of TLR4 on Mon1 and Mon3 in 65 patients with AMI, UAP, or SAP. The expression levels of TLR4 on Mon3 were lower in the UAP group than in the AMI group. Regarding correlations with other clinical indicators, Shan et al. (61) divided patients with UAP into low-risk patients and medium-high-risk patients according to the GRACE (global registry of acute coronary events) score. They confirmed that medium-high-risk patients had higher absolute count of Mon2 and MPA levels of total monocytes. Ikejima et al. (62) compared differences in peripheral blood leukocytes among patients with UAP and SAP and healthy controls and applied OCT results to divide UAP patients into a plaque rupture group and an unruptured group to study the relationship between monocyte heterogeneity and plaque stability. The results indicated that the number of CD14^+^CD16^+^CX3CR1^+^ monocytes in the UAP plaque rupture group was greater than that in the other 3 groups, and multiple logistic regression analysis revealed that the number of CD14^+^CD16^+^CX3CR1^+^ monocytes was an independent predictor of the presence of coronary plaque rupture. A similar conclusion was reached in the study by Imanishi et al. (63), which revealed that the percentage of circulating CD14^+^CD16^+^CX3CR1^+^ monocytes in patients with UAP was positively correlated with the percentage of fibrous caps, suggesting that CD14^+^CD16^+^CX3CR1^+^ monocytes may play an important role in the vulnerability of coronary artery plaques. Coincidentally, Jianhui Zhuang (60) also demonstrated that higher proportions and counts of the Mon2 subset in UAP patients were related to a thin fibrous cap. These results suggest that monitoring the amount of Mon2 and the expression of the corresponding activating receptor may be beneficial in the diagnosis and prognostic assessment of UAP patients. The underlying mechanisms, however, warrant further investigation through basic experiments *in vivo* and *in vitro*.

## 4 Future prospects for the clinical research on monocyte heterogeneity

This article summarizes the application of monocyte heterogeneity in clinical studies of SCAD and ACS. These studies have primarily focused on differences in monocyte heterogeneity, including the absolute number and proportion of subsets, the expression of functional receptors of subsets in different types of disease, and the associations of monocyte heterogeneity with clinical indicators and other indicators of disease (**Table 1**). Next, we discuss the highly significant yet frequently overlooked aspects of clinical studies related to monocyte heterogeneity in the field of CAD and propose rational prospects for future research directions.

**Table 1:** List of monocyte heterogeneity in different types of CAD.

Ref	Disease	Monocytes subsets	Heterogeneity of quantity and proportion	Heterogeneity of functional receptors expression	Heterogeneity related to clinical characterization	Cohort size
(19)	**SCAD**	Mon1, Mon2 and Mon3	the proportion of Mon1↓, Mon2 and Mon3↑(VS control)	EMMPRIN↑on Mon1 in all patients; EMMPRIN ↓ on all monocyte subsets in SCAD patients(VS AMI).		80 SCAD, 49 AMI and 34 healthy controls
(20)		Mon1, Mon2 and Mon3	the proportion of Mon1 ↑; proportion and absolute numbers of Mon3↓(VS the HBD group)	MPA ↑CCR5+ ↓of three subsets(VS the HBD group);Mon2 is the majority subset of CCR5 expression level	the number↓ of CCR5+ monocytes in CAD patients treated with statins (VS no treatment)	64 healthy blood donors (HBDs) and 60 patients with SCAD
(22)		CD14+CD16- and CD14+CD16+	the proportion ↑of CD14+CD16+in SAP (VS AMI)	the positive expression rate of TLR4↓on CD14+CD16+ in SAP(VS AMI)	TLR overexpression on CD14+CD16+ monocytes was associated with the pathogenesis of AMI.	22 AMI, 16 UAP,27 SAP, 15 healthy controls.
(23)		Mon1, Mon2 and Mon3	the proportion ↑of TLR4+ Mon2 in unstable plaque group(VS stable plaque)	the expression ↑of TLR4+in Mon2(VS other subsets)	TLR4+Mon2 was positively correlated with MMP-9 level and plaque vulnerability	65 patients with SAP who underwent MDCT
(24)		Mon1, Mon2 and Mon3	high Mon3 and low Mon1 presented impaired endothelial function	the expression↑ of CD11c on Mon3 (VS Mon1 and Mon2)	CD11c highly expressed on Mon3 associated with more advanced endothelial dysfunction	130 patients with CAD undergoing coronary artery bypass grafting
(25)		CD14+CD16-and CD14+CD16+	the proportion↑ of CD14+CD16+ in patients with vulnerable plaques(VS with no vulnerable plaques patients or healthy subjects)		the proportion of CD14+CD16+ monocytes was positively correlated with remodeling index and negatively correlated with CT attenuation value.	73 patients with SAP who underwent MDCT.
(26)		CD14+CD16-and CD14+CD16+	The frequency↑ of CD14+CD16+ in patients who had future coronary events(VS those who did not)		the frequency of CD14+CD16+monocytes was an independent predictor for future coronary events	271 patients who were suspected to have either SAP or silent myocardial ischemia and underwent coronary angiography
(27)		CD14+CD16- and CD14+CD16+	the proportion and number↑of CD14+CD16+ in patients with MVD (vs those with SVD or without CAD)		the proportion and number of CD14+CD16+ positively correlated with Gensini score	125 SAP and divided into 3 groups: without CAD,SVD, MVD.
(32)		Mon1, Mon2 and Mon3		the expressionof PSGL-1 on Mon2 in SAP↑ (VS contro),↓(VS AMI)	PSGL-1 on Mon2 monocytes may be a crucial role in plaque rupture and thrombus formation.	25 AMI, 20 UAP,35 SAP,20 control subjects
(71)		Mon1, Mon2 and Mon3	monocyte subset distribution was not significantly different in patients with and without statins		the number of Mon1 correlated with circulating PCSK9 level, the number of Mon3 inverse correlation with PCSK9 level in patients on statin treatmen	69 patients with SCAD
(79)		Mon1, Mon2 and Mon3			Mon2 monocyte counts were independently associated with plaque vulnerability,and correlated with the MAGE score in the non-DM patients	51 patients with asymptomatic CAD including 22 DM patients and 29 non-DM patients
(32)	**ACS**	Mon1, Mon2 and Mon3		the expression ↑of PSGL-1 on Mon2(VS other two subsets)	the expression of PSGL-1 on Mon2 monocytes may be a crucial role in plaque rupture and thrombus formation.	25 AMI, 20 UAP,35 SAP,20 control subjects
(33)		Mon1, Mon2 and Mon3			the proportion of Mon2 monocyte independently associated with PAI-1 activity in ACS	50 STEMI, 47 NSTEMI, 40 CA and 39 controls.
(19)	**AMI**	Mon1, Mon2 and Mon3	the proportion of Mon1↓, Mon2 and Mon3↑(VS control)	the expression of CD147 ↑on Mon1(VS Mon2 or Mon3);↑on all subsets of AMI patients(VS CAD)		80 SCAD, 49 AMI,34 healthy controls.
(22)		CD14+CD16+ and CD14+CD16-	the proportion↓ of CD14+CD16+ (VS SAP or UAP)	the expression of TLR4 on CD14+CD16+↑(VS CD14+CD16-subset);↑in AMI(VS other 3 groups)	TLR overexpression on CD14+CD16+ monocytes was associated with the pathogenesis of AMI.	22 AMI, 16 UAP,27 SAP, 15 healthy controls.
(32)		Mon1, Mon2 and CD14+CD16+		The expression ↑of PSGL-1 on Mon2 in AMI (VS other 3 groups)	the expression of PSGL-1 on Mon2 is a crucial role in plaque rupture and thrombus formation.	25 AMI, 20 UAP,35 SAP,20 control subjects
(35)		Mon1, Mon2 and Mon3	the absolute number of Mon1↑transiently, Mon2 and Mon3↑continuously after ischemic injury	phagocytic and migratory activities ↑in AMI (VS control)		12 AMI and 8 healthy volunteers
(36)		Mon1, Mon2 and Mon3		the adhesion efficiency ↑of Mon2(VS Mon1 or Mon3 );↑in AMI (VS healthy subjects)	the elevated CD11c on Mon2 is associated with elevated clinical correlates of MI severity	26 healthy subjects,9 subjects for the fructose feeding study, and 18 fasting patients AMI
(37)		CD14+CD16+ and CD14+CD16-			the counts of CD14+CD16- was negative correlation with ΔCFVR in AMI patients	29 patients with primary anterior AMI whotreated using PCI
(38)		Mon1, Mon2 and Mon3			peak of Mon1 negatively correlated with myocardial salvage index,posotively correlated with MAGE	36 AMI patients
(39)		Mon1, Mon2 and Mon3	the absolute number ↑of Mon2 in the rupture patients(VS the non-rupture patients)		the number of Mon2 correlated positively with MAGE	24 patients with plaque rupture and 13 patients without plaque rupture
(42)	**NSTEMI**	CD14+CD16- and CD14+CD16+	the absolute number↑ of two subsets (VS control);Peak levels ↓of CD14++CD16- (VS STEMI )		the peak value of CD14++CD16- posotively correlated with peak CK and CK-MB levels; peak value of CD14+CD16+ posotively correlated with Gensini score	30 STEMI, 30 NSTEMI, 25 SAP
(43)		Mon1, Mon2 and Mon3	the absolute number ↑of all monocyte subsets(VS control)	the expression of CD11b ↑on all subsets, CX3CR1 ↓on Mon1 and Mon2 and HLA-DR↓on Mon2 and Mon3 (VS NC)		38NC(ACS negative,13UAP, 29NSTEMI, 42 STEMI
(47)		Mon1, Mon2 and Mon3	the relative frequency of each monocyte subset was similar in all subjects	the expression of CD11c ↑on all the monocytes,VLA-4↑ in Mon2 (VS CAD and healthy)and CX3CR1↑in total(VS healthy)	CD11c on Mon2 acts as a mechanoregulator to activates inflammatory in cardiac patients	56 SCAD, 46 NSTEMI,15 healthy subjects
(48)		CD14++ CD16+CCR2+ and CD14+ CD16++CCR2-	the absolute number ↑ of TLR4+CD14++ CD16+CCR2+ in NSTEMI(VS SCAD)		the counts of TLR4+CD14++ CD16+CCR2+ was correlated positively with Plasma IL-6 level	50 STEMI, 48 NSTEMI,40 SCAD
(53)		Mon1, Mon2 and Mon3		the expression of VCAM-1r ↑on Mon2 and Mon3,no change in ICAM-1r expression(VS SCAD)	the expression of VCAM-1r on Mon1 correlated positively with plasma IL-1β levels.	50 STEMI,48 NSTEMI,40 SCAD
(42)	**STEMI**	CD14++CD16- and CD14+CD16+	the peak levels↑ of two subsets and reached later in STEMI within 7 days after onset(VS NSTEMI or SAP)			30 STEMI, 30 NSTEMI, 25 SAP
(43)		Mon1, Mon2 and Mon3	the absolute number ↑of all monocyte subsets(VS NC group)	the expression of CD11b and CX3CR1 ↓on all subsets in STEMI(VS NC group)		38 NC(ACS negative),13UAP, 29NSTEMI, 42 STEMI
(48)		CD14++ CD16- CCR2+, CD14++ CD16+CCR2+ and CD14+ CD16++ CCR2-	the absolute number↑ of TLR4+CD14++ CD16+CCR2+ and TLR4+CD14++ CD16+CCR2+ (VS CAD)		the counts of TLR4+CD14++ CD16+CCR2+ correlated positively with Plasma IL-6 levels	50 STEMI,48 NSTEMI,40 SCAD
(49)		Mon1, Mon2 and Mon3	the absolute number ↑of Mon1 and Mon2 (VS SCAD or healthy)		the counts of Mon1 monocytes negatively correlated with cFLC	48 STEMI,40 SCAD,37 healthy
(50)		Mon1, Mon2 and Mon3	the number ↓ continuously of Mon1 and Mon2 for 7 days, Mon3 showed the opposite trend		the number of Mon1 and Mon2 inversely with LVEF,the number Mon3 correlated with BZ mass	102 patientsn with a first STEMI
(51)		Mon1, Mon2 and Mon3	Mon1 peaked at 12h and returned to nadir levels at 24h,Mon2 peaked at 24h,Mon3 peaked at 6h after injury		the number of Mon3 and their subsets expressing CCR2, CD42, and CD11b could be important predictors of clinical outcomes in patients with STEMI.	100 patients with STEMI who underwent PCI
(52)		CD14++ CD16- CCR2+, CD14++ CD16+CCR2+ and CD14+ CD16++ CCR2-	the counts of CXCR4+ and KDR+ monocytes↑ following MI; CXCR4+ CD14++ CD16+CCR2+monocytes↓during follow-up(VS SCAD )	the expression of CD163 ↓on CD14+ CD16++ CCR2- monocytes(VS SCAD )	the CD14++ CD16+CCR2+ subset has the most prominent role in the observed changes in reparative monocytes in MI.	50 STEMI,40 SCAD
(53)		Mon1, Mon2 and Mon3		the expression of VCAM-1r↓on all subsets,IL-6r ↓on Mon2 (VS SCAD)and IL-6r ↓on Mon1 and Mon2 during follow-up	the expression of VCAM-1r on Mon1 correlated positively with plasma IL-1β level.	50 STEMI,48 NSTEMI,40 SCAD
(54)		Mon1, Mon2 and Mon3	the absolute number↑of Dectin-1+Mon1 and Dectin-1+Mon2 monocyte(VS control )		the absolute number of Dectin-1+Mon1 and Dectin-1+Mon2 monocyte positively correlated with the severity of cardiac dysfunction	90 STEMI,45 control
(55)		CD14+CD16- CCR2+ and CD14+CD16+CX3CR1+	the absolute number↑of CD14+CD16+CX3CR1+ in patients with restenosis(VS patients without restenosis)		the counts of CD14+CD16+CX3CR1+ had positive correlation with angiographic late lumen loss	71 patients with AMI who underwent bare-metal stents
(56)		CD14++CD16-, CD14++CD16+CCR2+ and CD14+CD16++CCR2-	the counts↑ of 3 subsets in patient with MACE (VS no MACE)	the active of phagocytosis↑of CD14++CD16- subset in STEMI(VS other subsets)	the counts of CD14++CD16+CCR2+ were independently predictive of MACE	245 STEMI patients
(57)		CD14+CD16+ and CD14+CD16-	The peak levels ↑of CD14+CD16- in patients with MVO(VS without MVO)		Post-reperfusion enhancement of CD14+CD16-monocytes was associated with MVO in patients with STEMI	72 patients with primary STEMI successfully treated with stenting
(22)	**UAP**	CD14+CD16- and CD14+CD16+	the proportion↑ of CD14+CD16+(VS AMI)	the positive expression rate↓ of TLR4 on CD14+CD16+monocytes (VS AMI)	the overexpression of TLR on CD14+CD16+monocytes was associated with the pathogenesis of AMI.	22 AMI, 16 UAP,27 SAP, and 15 healthy controls
(32)		Mon1, Mon2 and Mon3		the expression of PSGL-1 on Mon2↑(VS control),but ↓(VS AMI)		25 AMI, 20 UAP,35 SAP,20 control subjects
(43)		Mon1, Mon2 and Mon3	the number ↓ of Mon1 (VS NSTEMI)	the expression of CD11b on mon1↑(VS STEMI) on mon2 or mon3 ↓(VS NSTEMI)		38 NC(ACS negative),13UAP, 29NSTEMI, 42 STEMI
(60)		Mon1, Mon2 and Mon3	the proportion and counts of Mon2↑(VS control)and↑ in UAP patients with TCFA(VS patients without TCFA)		Circulating Mon2 appears to be a promising marker for the severity of atherosclerotic plaque	48 UAP,31 STEMI,33 healthy control
(61)		Mon1, Mon2 and Mon3	the counts↑of Mon2 and Mon3(VS SCAD), the counts↑of Mon2 in UAP with intermediate-to-high risk patients (VS with GRACE score-determined low risk patients)		the up-regulation of Mon2 counts, Mon2-associated MPAs and total MPAs level were independently associated with GRACE score.	30 SCAD,95 UAP
(62)		CD14++CD16+ and CD14+CD16-	the counts↑ of CD14+CD16+CX3CR1+in plaque rupture patients(VS other 3 groups)		the counts of CD14+CD16+CX3CR1+ monocytes were independent predictors of the presence of ruptured plaque	46UAP including 27 rupture and 19 non-rupture,30 SAP, 25healthy
(63)		CD14+CD16-CCR2+ and CD14+CD16+CX3CR1+	the proportion ↓ of CD14+CD16+CX3CR1+ monocytes in patients with statin treatmen(VS without)		the percent changes in CD14+CD16+CX3CR1+ monocytes were negative correlation with FCT and related to levels of CRP	40UAP

Mon1, CD14++CD16-monocyte; Mon2, CD14++CD16+ monocyte; Mon3, CD14+CD16++monocyte; CAD, coronary artery disease; SCAD, stable coronary artery disease; ACS, acute coronary syndrome; AMI, acute myocardial infarction; STEMI, ST-segment elevation myocardial infarction; NSTEMI, Non-ST-elevation myocardial infarction; UAP, unstable angina pectoris; SAP, stable angina pectoris; MACE, major adverse cardiovascular events; MDCT, multidetector computed tomography; SVD, single-vessel disease; MVD, multiple-vessel disease; CRP,C-reactive protein; MPA, Monocyte Platelet Aggregate;TLR4, Toll-like receptor 4; PSGL-1,P-selectin glycoprotein ligand-1; PCSK9, proprotein convertase subtilisin/kexin type-9; DM, diabetes mellitus; MVO, microvascular obstruction; FCT, fibrous cap thickness; VH-TCFA, virtual histology thin-cap fibroatheroma.

### 4.1 The role of monocyte heterogeneity in explaining the contradictory results of clinical studies

Understanding monocyte heterogeneity can not only help us understand the overall picture of coronary heart disease but also explain some clinical contradictions. The negative association between low-density lipoprotein cholesterol (LDL-C) and clinical outcomes following myocardial infarction is referred to as the lipid paradox (64). Dong et al. (65) discussed this phenomenon from the perspective of circulating monocyte heterogeneity and reported that the level of LDL-C at admission was negatively correlated with the number of circulating Mon2 cells in patients with STEMI. After patients were categorized according to the cut-off values of 85 mg/dL for LDL-C and the median for Mon2, low LDL-C-associated MACE risk was observed only in those with high Mon2 counts. These results provide a reasonable explanation for the “lipid paradox” from the perspective of monocyte heterogeneity. Similar contradictions are also evident in high-density lipoprotein (HDL) levels. Small HDL from healthy subjects has been shown to have strong atherosclerotic protective effects, including the potential to increase cholesterol efflux, as well as stronger antioxidant and anti-inflammatory activities. However, clinical studies have shown that the level of small HDL is positively correlated with the severity of atherosclerotic disease, whereas the level of large HDL is inversely correlated with disease severity. Krychtiuk et al. (66) suggested that this may be related to monocyte heterogeneity. They reported that the level of small HDL was positively correlated with the percentage of Mon3 monocytes and negatively correlated with Mon1 monocytes, suggesting a nonnegligible relationship between lipoprotein function and monocyte heterogeneity. This result explains why the level of small HDL, which should play a protective role in atherosclerosis, is positively correlated with the existence and severity of atherosclerosis. With the deepening of basic and clinical research on CAD, many contradictory findings may emerge in the future. At this time, monocyte heterogeneity, which is an important factor throughout the disease course, should be considered.

### 4.2 Focusing on monocyte heterogeneity in the process of clinical diagnosis and treatment

Monocyte heterogeneity can be used to evaluate the risk of adverse cardiovascular events not only in patients with CAD but also in patients without cardiovascular disease. For example, patients with psoriasis have been shown to have an increased risk of death due to cardiovascular disease, which is associated with an increase in the number of Mon2 monocytes (67). In addition, the increased risk of myocardial infarction in patients with human immunodeficiency virus (HIV) infection receiving combined antiretroviral therapy (ART) is inextricably linked to an increase in the number of Mon1 monocytes (68). Therefore, monitoring changes in monocyte heterogeneity in patients without cardiovascular diseases during diagnosis and treatment is helpful for evaluating their risk of cardiovascular complications.

Monocyte heterogeneity is affected by therapeutic drugs for CAD. Ozaki et al. (69) showed that patients treated with aliskiren combined with angiotensin converting enzyme inhibitors (ACEI)or angiotensin receptor blocker (ARB) drugs achieved greater improvement in the degree of myocardial salvage after AMI, and the absolute number and proportion of Mon1 cells were lower than those in patients who used only ACEI or ARB drugs. This finding suggests that the combination of aliskiren with ACEI or ARB drugs in improving myocardial salvage may be related to the reduced peak Mon1 levels. The efficacy of statins in affecting monocyte heterogeneity has also been preliminarily reported (70). The percentage of CD14^+^CD16^+^CX3CR1^+^ monocytes in UAP patients treated with statins was previously reported to be lower than that in patients without statins (63), and the number of CCR5^+^ monocytes in CAD patients decreased significantly among the 3 subsets (20). Proprotein convertase subtilisin/kexin type-9 (PCSK9) is an enzyme that promotes the degradation of low-density lipoprotein receptor (LDL-R) in hepatocytes. The inhibition of PCSK9 has emerged as a novel target for lipid-lowering therapy. Krychtiuk et al. (71) demonstrated that the level of circulating PCSK9 in patients with SCAD after statin treatment was positively correlated with the number of Mon1 cells but inversely correlated with Mon3 cell number, suggesting a new link between PCSK9 regulation, innate immunity, and atherosclerotic disease in statin-treated patients. In brief, exploring the molecular mechanism of monocyte heterogeneity contributes to comprehensive analyses of the pharmacological effects of drugs.

In addition, some studies have shown that exercise can promote human anti-inflammatory abilities by altering monocyte heterogeneity (72, 73). A meta-analysis (74) confirmed that exercise-based cardiac rehabilitation (CR) can reduce cardiovascular mortality and the recurrence rate of cardiac events and improve the health and outcomes of patients with coronary heart disease under contemporary medical management. To explore the relationship between exercise-based CR and monocyte heterogeneity, we not only explored the role of monocyte heterogeneity in the field of CAD but also provided more effective evidence for exercise-based CR.

### 4.3 Making full use of scRNA-seq to explore the impact of the functional heterogeneity of monocytes on diseases

Although the phenotypic heterogeneity of monocytes grouped by CD14 and CD16 offer important guiding significance, the characteristics of monocyte heterogeneity in diseases cannot be fully reflected. With respect to the functional heterogeneity of monocytes, scholars have focused on the expression of functional receptor molecules and secretory factors of monocytes and their interactions with other cell-derived ligand molecules or secretory factors (75–77). To reflect the functional characteristics of different subsets, functional labels are often affixed to specific subsets, such as “proinflammatory monocytes” and “anti-inflammatory monocytes”. This labelling of cell subsets by several receptor molecules is one-sided and not sufficient to reflect the complexity of monocyte heterogeneity in the progression of diseases. Schmidl et al. (78) performed a transcriptomic analysis of 3 subsets of peripheral blood monocytes from healthy subjects and revealed the metabolic heterogeneity of monocyte subsets: Mon1 expressed higher levels of carbohydrate metabolism genes in preparation for anaerobic energy production, whereas Mon2 expressed higher levels of genes related to the mitochondrial oxidative pathway. Single cell RNA sequencing (scRNA-seq) has been widely used in clinical research in recent years. The introduction of scRNA-seq technology into the clinical study of CAD will help us explore more functional receptor molecules and more detailed classifications of monocyte subsets associated with the clinical phenotype. In addition, the interactions between monocyte heterogeneity and other cells cannot be overlooked in the development of CAD, and the effects of monocyte heterogeneity and platelet communication on this disease have been studied (20, 61). However, the consequences of the interactions between monocyte heterogeneity and T cells, B cells, and other immune cells in this disease are unclear, and are worthy of further exploration. Information on intercellular interactions provided by scRNA-seq technology can facilitate exploration in clinical research and provide opportunities to solve the above problems.

### 4.4 Deep mining of imaging information is helpful for fully reflecting the application value of monocyte heterogeneity

In clinical practice, many scholars have associated conventional imaging data with monocyte subsets by, for example, evaluating plaque stability via IVUS or MDCT and exploring the clinical potential of circulating monocyte heterogeneity (19, 23, 56, 60, 63, 79). The limitation of clinical studies is the difficulty of obtaining coronary plaque tissue, and OCT can provide detailed characteristics of plaques *in vivo*, which allows analyses of the associations between circulating monocyte heterogeneity and plaque tissue characteristics. For example, OCT can quantify macrophage infiltration in plaques (80, 81). Future studies can use OCT technology to examine the infiltration of macrophages and evaluate the association between monocyte heterogeneity in peripheral blood and macrophage heterogeneity in plaque tissue. During vascular intervention, coronary angiography and intravascular ultrasound (IVUS) are considered the “gold standard” for the diagnosis of CAD, but these 2 methods can evaluate only the degree of stenosis. Pijls et al. (82) proposed a new index, the fractional flow reserve (FFR), to calculate coronary blood flow through pressure measurement. After long-term basic and clinical research, the FFR has become a recognized index for the functional evaluation of coronary artery stenosis (83). Nonetheless, no studies have explored the relationship between the FFR and peripheral blood monocyte heterogeneity. Indeed, much important imaging information has been overlooked in the process of clinical research. Hence, in-depth exploration of imaging information will help to fully reflect the application value of monocyte heterogeneity in CAD.

### 4.5 Undeveloped mineral deposits in the study of monocyte heterogeneity: shear stress stimulation

David Julius and Ardem Patapoutian won the 2021 Nobel Prize in Physiology or Medicine for the discovery of temperature and tactile receptors (84). When the human body senses an external physical stimulus, in addition to visible behavioral changes, cells also make corresponding biological behavior changes, such as those observed from the effects of blood flow shear stress on endothelial cells and exercise on monocytes. In the atherosclerotic pathological microenvironment, mechanoreceptors on the apical surfaces of endothelial cells can sense the force exerted by blood flow on the vascular wall, that is, wall shear stress (WSS), and induce cellular and molecular responses (85). In the early stage of atherosclerosis, monocytes adhere to endothelial cells, and “patrolling” the endothelial surface is a prerequisite for monocytes to enter the lesion area. Although WSS does not directly act on the membrane surface of circulating monocytes, WSS is bound to affect the heterogeneity of monocytes. At present, few related studies in this area are available. Only one *in vitro* study (86) suggested that WSS could promote the secretion of IL-1β by monocytes infected with *Streptococcus pneumoniae*, which preliminarily confirmed that WSS produced in the blood could affect the functional heterogeneity of monocytes. With the further development of related research, the effect of shear stress on monocytes is expected to be gradually clarified, and new mechanoreceptor may be found such that we can gain a deeper understanding of the role of monocytes in CAD.

## 5 Conclusion

In summary, circulating monocyte heterogeneity plays a significant and extensive role in the clinical diagnosis, treatment, and prognosis of CAD. However, the current classification scheme does not fully reflect the heterogeneity of circulating monocytes in patients with CAD. The first part of this article comprehensively addresses the timeliness and complexity of monocyte heterogeneity in different types and stages of CAD. Furthermore, substantial variations in results may arise due to different sampling times and selection of control groups. With the widespread implementation of high-parameter flow cytometry and scRNA-seq sequencing, it is anticipated that more functional receptor molecules and more detailed monocyte subsets with high specificity to the clinical phenotype of the disease will be identified. By rigorously standardizing the selection of research subjects, establishing appropriate control groups, utilizing scRNA-seq techniques judiciously, thoroughly analyzing imaging information, and comprehensively integrating clinical data, the translational and applicative value of circulating monocyte heterogeneity in the field of CAD will be enhanced.
